# Supplementary material for: Tannery Wastewater Recalcitrant Compounds Foster the Selection of Fungi in Non-Sterile Conditions: A Pilot Scale Long-Term Test
Source: Int J Environ Res Public Health. 2021 Jun 11;18(12):6348. doi: 10.3390/ijerph18126348 (PMC8296185; doi:10.3390/ijerph18126348)
Supplement: Supplementary file 1 [file ijerph-18-06348-s001.zip › ijerph-1249692-supplementary.pdf]

**Supplementary materials:**

# Tannery wastewater recalcitrant compounds foster the selection of fungi in non-sterile conditions: a pilot scale long-term test

Francesco Spennati[a*], Salvatore La China[b], Giovanna Siracusa[c], Simona Di Gregorio[c], Alessandra Bardi[f], Valeria Tigini[d], Gualtiero Mori[a], David Gabriel[e], Giulio Munz[f].

[a] Cer$^2$co, Consorzio Cuoiodepur, Via Arginale Ovest 81, 56020, San Romano - San Miniato, Pisa, Italy.

[b] Department of Life Sciences, University of Modena and Reggio-Emilia, Via Giuseppe Campi 287, 41125, Modena, Italy.

[c] Department of Biology, University of Pisa, Via Luca Ghini 13, 56126, Pisa, Italy.

[d] MUT, Department of Life Sciences and Systems Biology, University of Turin, Viale Mattioli 25, 10125, Torino, Italy.

[e] GENOCOV, Department of Chemical, Biological and Environmental Engineering, School of Engineering, Autonomous University of Barcelona, 08193, Bellaterra, Barcelona, Spain.

[f] Department of Civil and Environmental Engineering, University of Florence, Via Santa Marta 3, 50139, Firenze, Italy.

## Materials and Methods

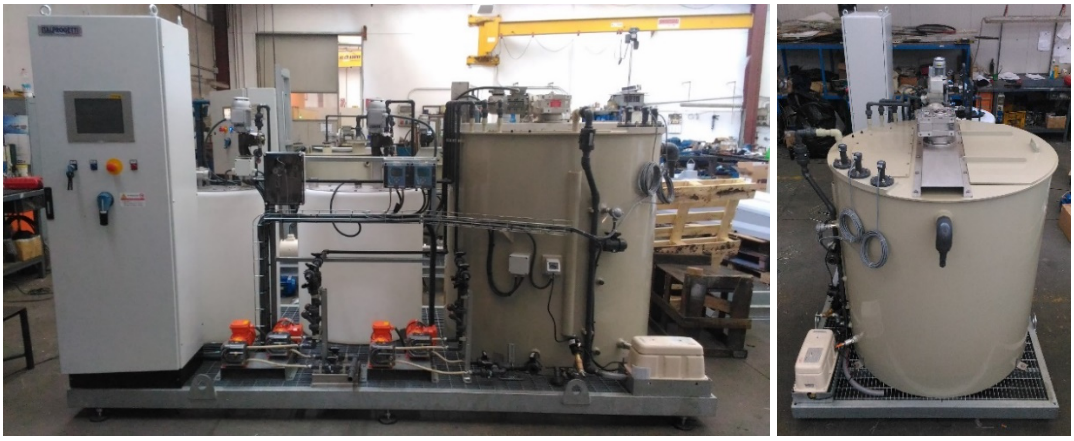

**Figure S1.** Lateral view of the pilot reactor (**left**) and front view of the pilot reactor (**right**).

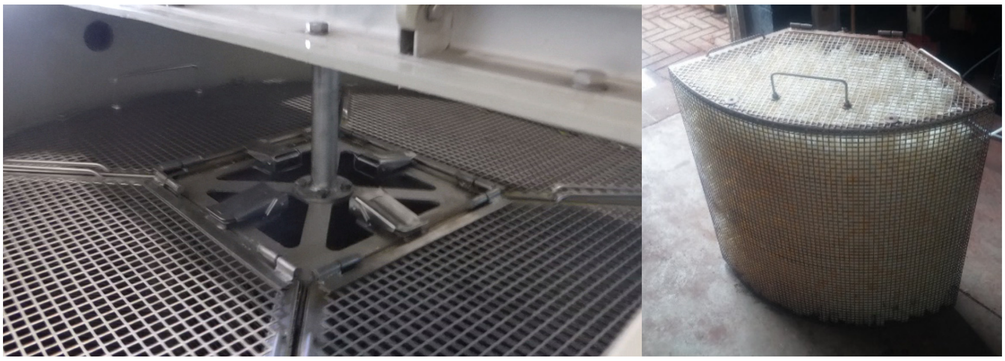

**Figure S2.** Internal view of the rotating cage (**left**) and one sector full of PUF cubes (**right**).

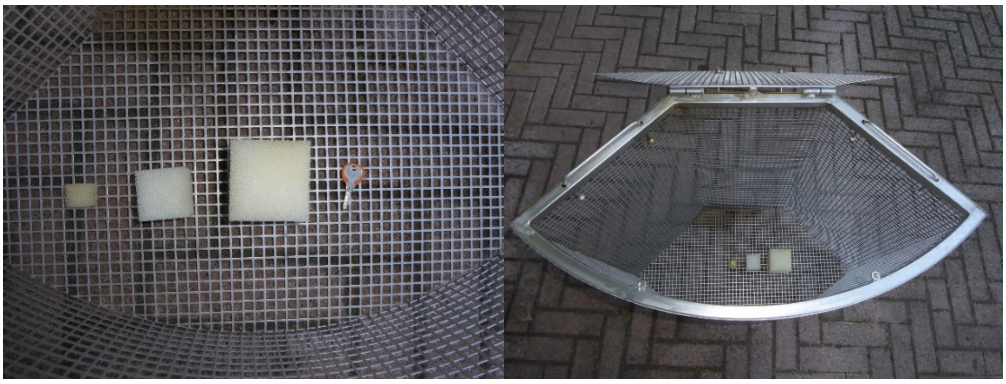

**Figure S3.** Representative pictures of the three sizes of PUF cubes (**left**) and one sector of the cage (**right**).

**Table S1.** Pilot-scale reactor: main sensors and actuators installed.

| **Actuators** | | | | |
|---|---|---|---|---|
| **P&I** | **Description** | **Supplier** | **Model** | **n** |
| P02 | Vertical mixer | Italprogetti | Engine 63 | 1 |
| G11A/B | Peristaltic pump | Watson marlow | 521F/R2C | 2 |
| G21A/B | Peristaltic pump | Watson marlow | 521F/R2C | 2 |
| P31 | Air compressor | Techma gp | LAM200 | 1 |
| G43 | Dosing pump | Obl | RBB 30 P 95 | 1 |
| P52 | Vertical mixer | Italprogetti | Engine 71 | 1 |
| G53 | Dosing pump | Obl | RBB 30 AC 95 | 1 |
| EV04 | Valve | Omal | GF3/4″ | 1 |
| **Sensors** | | | | |
| DOIC03 | DO probe | Hach | LXV416.99.20001 | 1 |
| PHIC04 | pH probe | Hach | DPD1R1.99 | 1 |
| RXIC05 | Redox probe | Hach | DRD1R5.99 | 1 |
| TIC06 | Temperature probe | Seico | PT 100 | 1 |

**Table S2.** Characteristic of the sectors of the pilot-scale reactor.

| | Volume ($m^3$) | Surface ($m^2$) | Dry Mass (kg) | Dry Mass/Volume ($kg\ m^{-3}$) |
|---|---|---|---|---|
| **Sector 1** | 0.094 | 56 | 0.468 | 4.99 |
| **Sector 2** | 0.078 | 47 | 0.377 | 4.86 |
| **Sector 3** | 0.072 | 43 | 0.243 | 3.39 |
| **Sector 4** | 0.094 | 56 | 0.468 | 4.99 |
| **Pilot bioreactor** | 0.337 | 202 | 1.556 | 4.56 |

**Table S3.** Characterisation of tannin-rich effluent collected from a tannery factory.

| **Parameter** | **Unit** | **Value** |
|---|---|---|
| pH | pH | 3.6 |
| sCOD | $mg\ O\ L^{-1}$ | 64,331 |
| DOC | $mg\ C_{org}\ L^{-1}$ | 14,546 |
| Total N. | $mg\ N\ L^{-1}$ | 1,071 |
| TSS | $mg\ TSS\ L^{-1}$ | 12,540 |
| VSS | $mg\ VSS\ L^{-1}$ | 9,050 |
| Chlorides | $mg\ Cl\ L^{-1}$ | 13,983 |
| Sulphates | $mg\ S\ L^{-1}$ | 5,369 |
| Sulphides | $mg\ S\ L^{-1}$ | 120 |

| | | |
|---|---|---|
| Nitrites | mg N $L^{-1}$ | 4.7 |
| Nitrates | mg N $L^{-1}$ | 0.5 |
| Ammonium | mg N $L^{-1}$ | 834.7 |
| Conductivity | mS $cm^{-1}$ | 45.3 |
| **Metals** | | |
| Chromium | mg $L^{-1}$ | 0.4 |
| Iron | mg $L^{-1}$ | 15.8 |
| Cadmium | mg $L^{-1}$ | < 0.2 |
| Lead | mg $L^{-1}$ | < 0.2 |
| Zinc | mg $L^{-1}$ | 3.2 |
| Aluminium | mg $L^{-1}$ | 32.2 |
| Copper | mg $L^{-1}$ | 0.6 |
| Boron | mg $L^{-1}$ | 0.4 |

## Results and Discussion

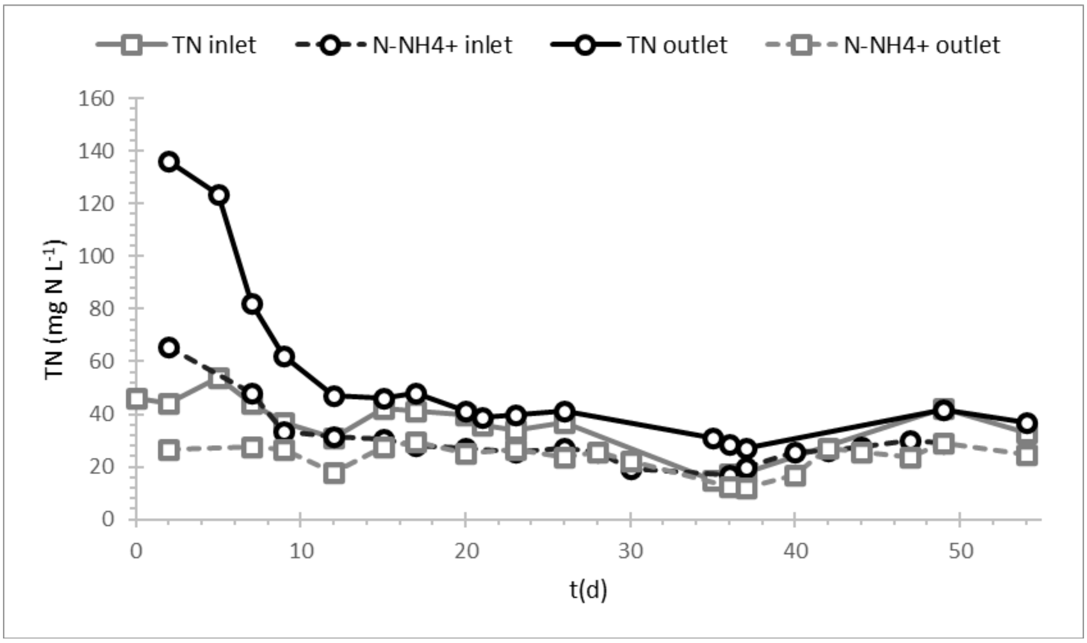


**Figure S4.** Total nitrogen and ammonium concentration inlet and outlet in the pilot reactor during the start-up phase. Noteworthy, it was observed a released of nitrogen (part detected as ammonium) from the reactor in the first two weeks. The release of nitrogen during the first two weeks could be related to the residuals of PUF production process possibly present on its surface, in fact, in the industrial PUF production process, isocyanate reacts with an amine leading to the synthesis of urea (double amide of the acid carbonate) as byproduct.

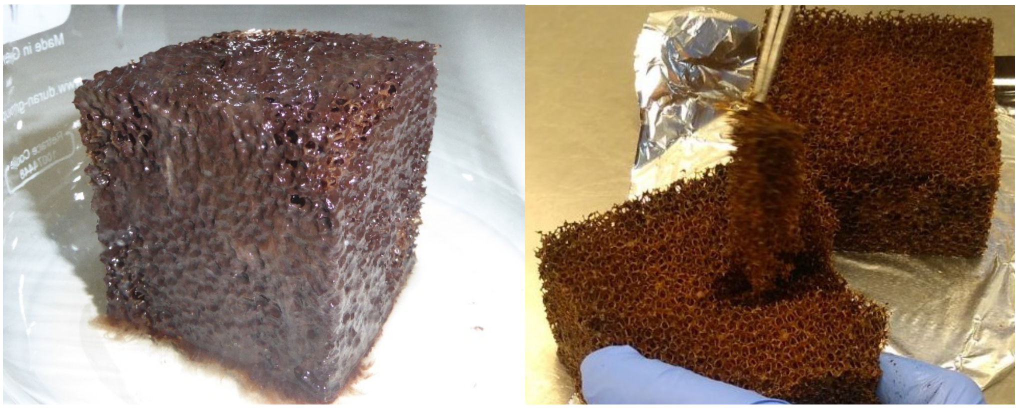

**Figure S5.** PUF cubes with 7.5 cm size: external view (left) and internal view (right).

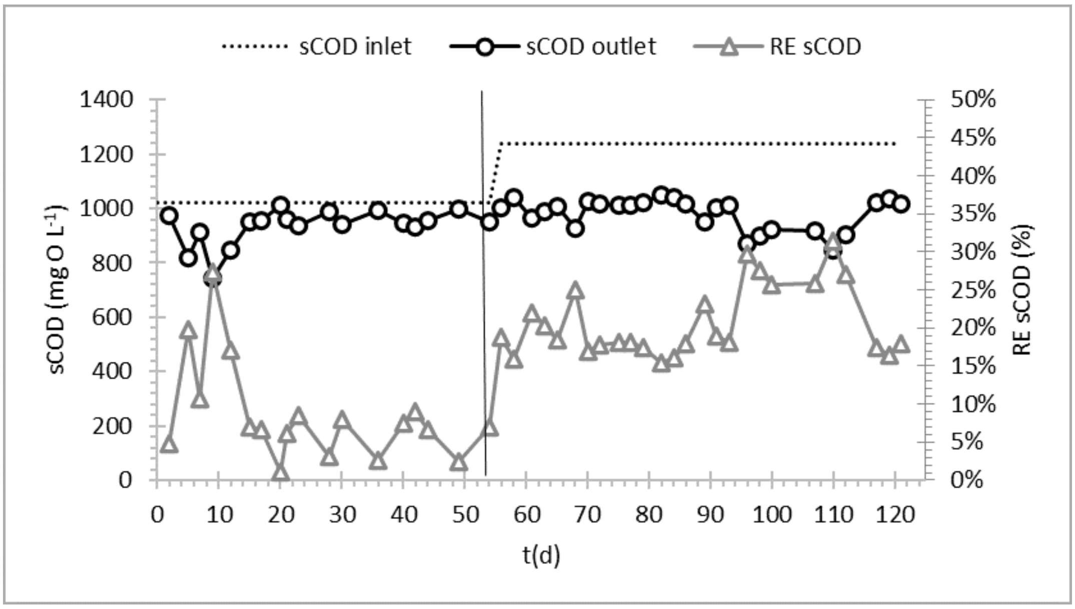


**Figure S6.** Inlet and outlet sCOD and sCOD removal percentage during continuous treatment in the pilot reactor. Vertical line divided the start-up phase from the treatment phase.

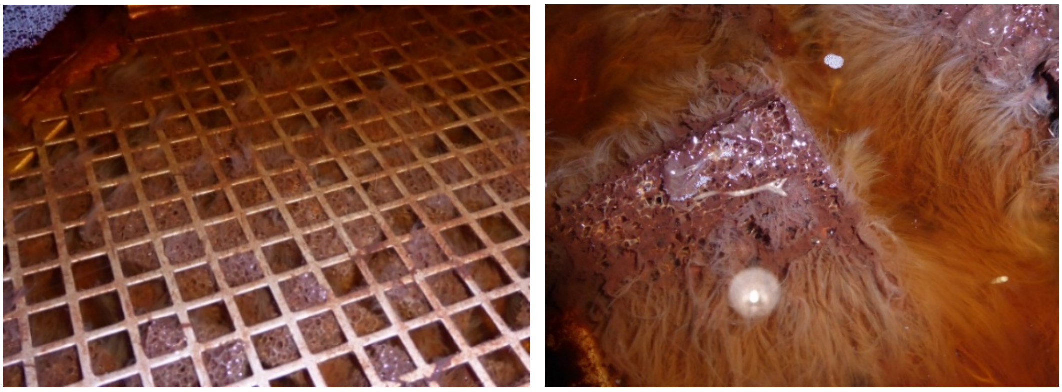

**Figure S7.** Pictures of immobilised PUF cubes in the pilot at the end of the start-up phase. On the left a picture of PUF cubes located in the middle of the sector (less exposed to the share stress of rotation) on the right a detail of a PUF.

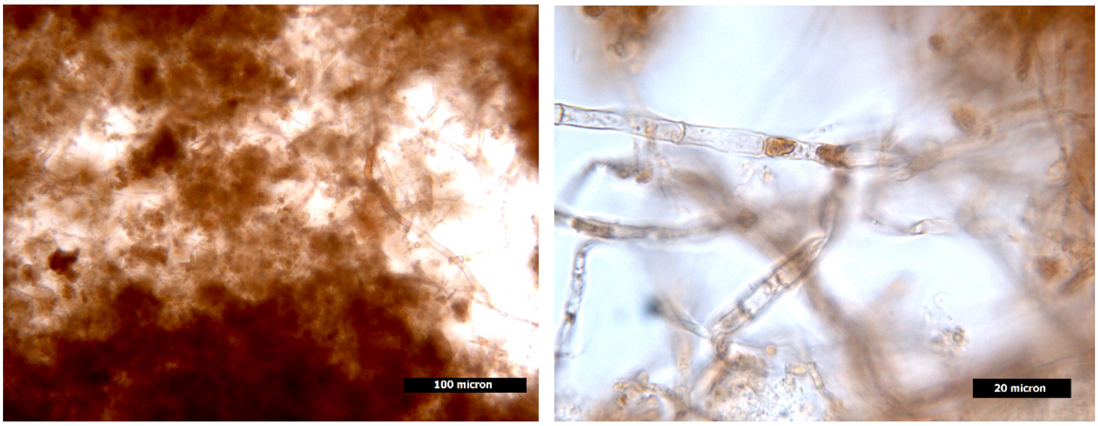


**Figure S8.** Microscope pictures of biofilm from immobilised PUF cubes in the pilot at the end of the start-up phase.

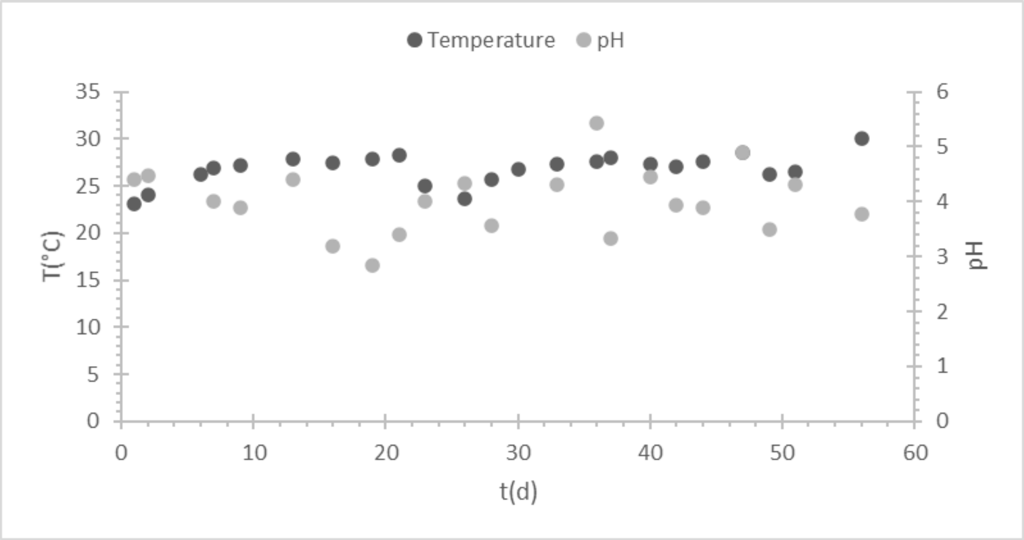


**Figure S9.** The average temperature and pH during the start-up in the pilot-scale reactor.

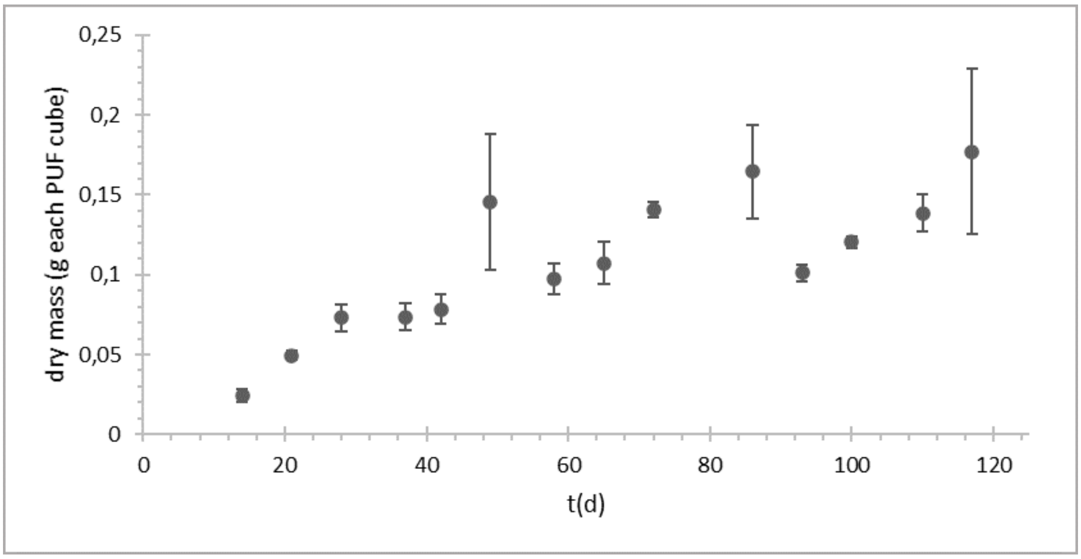


**Figure S10.** Dry mass in PUF cubes collected from the pilot reactor. Values are given as average among three replicates +/- Standard Deviation (SD).

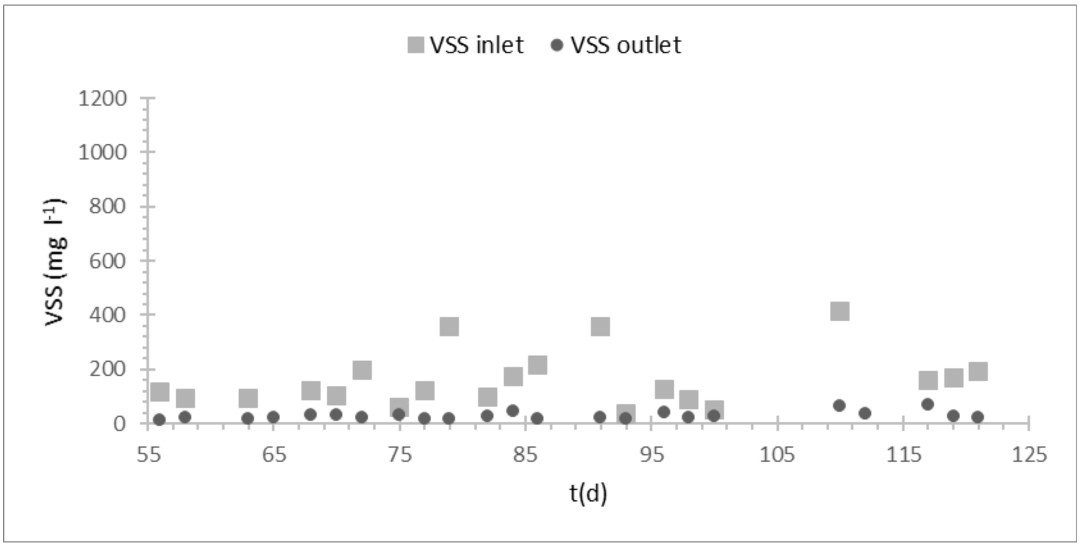


**Figure S11**. VSS inlet and outlet in the pilot reactor during the treatment

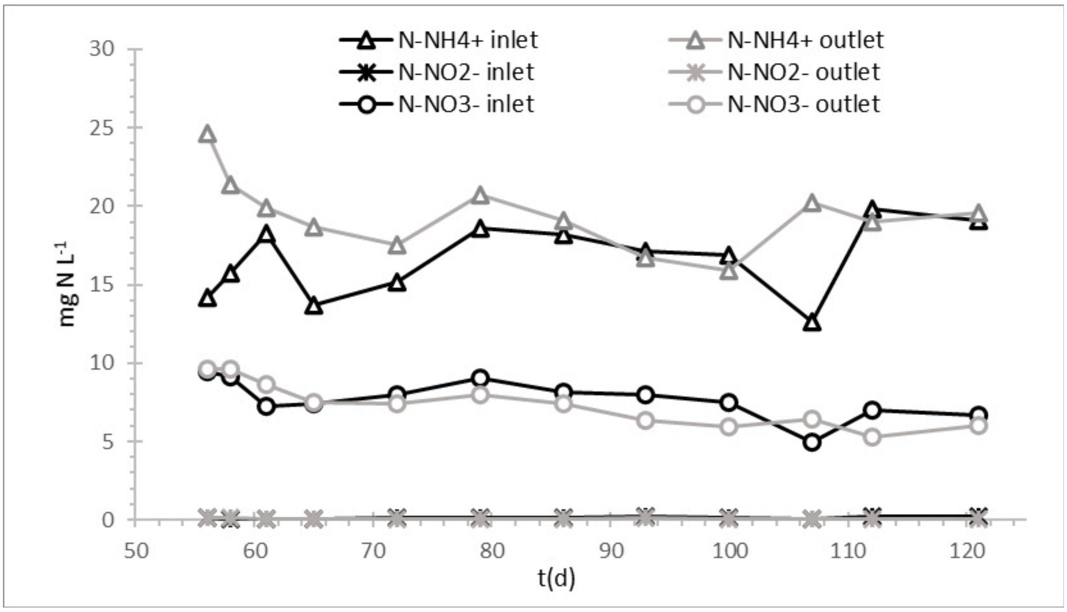


**Figure S12.** Ammonium, Nitrate and Nitrite concentration inlet and outlet in the pilot reactor during the treatment phase.

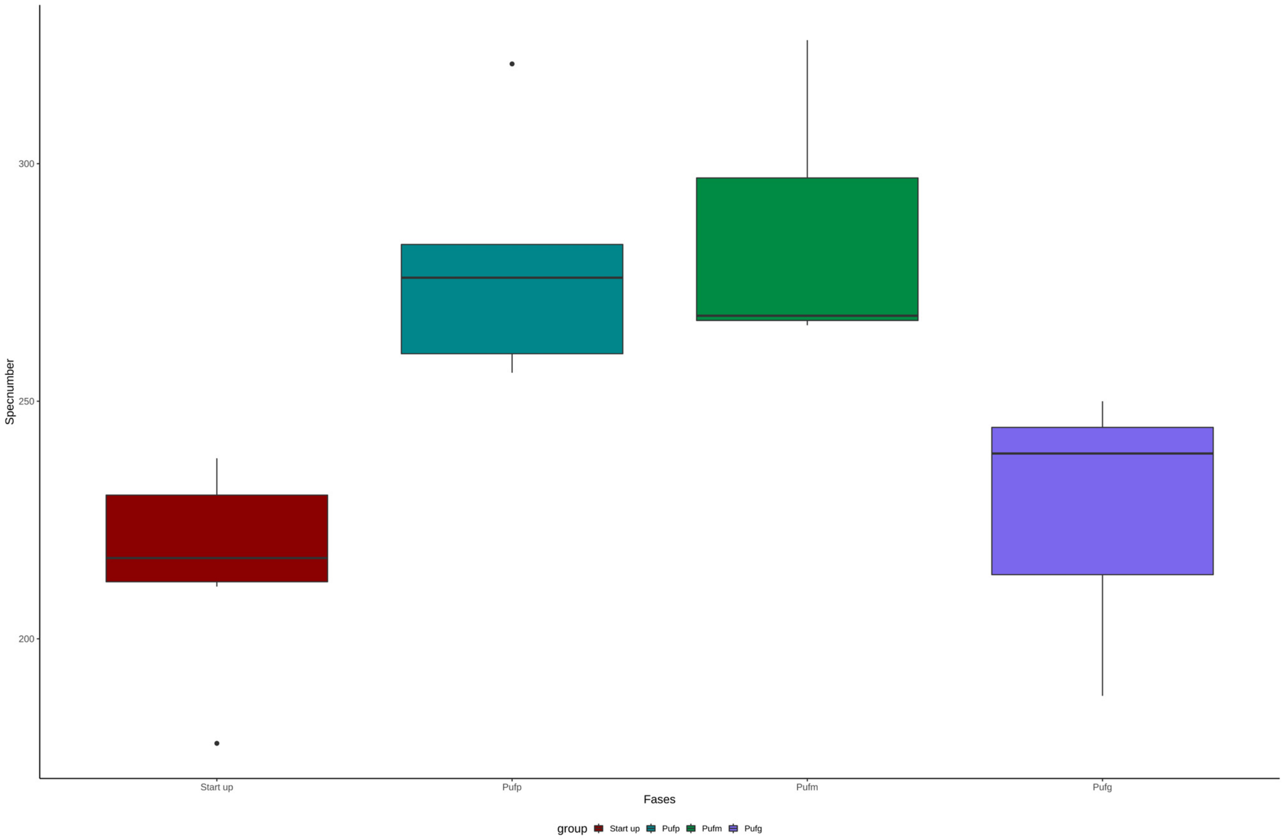


**Figure S13.** Number of species.

**Table S4.** Characteristic Alpha diversity indices among the phases. The groups are indicated by letters. Groups with the same letters are not statistically significant different ($p < 0.05$). Values are gives as average among replicates ± Standard Deviation (S.D.).

| | Start up | Pufp | Pufm | Pufg |
|---|---|---|---|---|
| Species nr. | 215.83±21.39(b) | 279.20±25.88(a) | 286.67±34.08(a) | 225.67±33.08(b) |
| Simpson | 0.69±0.05(a) | 0.72±0.06(a) | 0.72±0.01(a) | 0.73±0.02(a) |

**Table S5.** SIMPER analysis. The upper part of the table shows the percentage of difference among pairwise comparisons among phases. The lower part of the table reports the families that contribute to these differences with the respective percentages. Only the statically significant differences are reported ($p < 0.05$).

| | **Inoculum** | **Start up** | **End Treatment** |
|---|---|---|---|
| **Inoculum** | | 6.2% | 7.5% |
| **Start up** | *Xanthomonadaceae* (6.2%) | | 39.4% |
| **End treatment** | *Xanthomonadaceae* (7.53%) | *Xanthomonadaceae* (13.5%)<br>*Rhabdochlamydiaceae* (8.01%)<br>*Acidobacteriaceae* (4.83%) | |

**(A)**

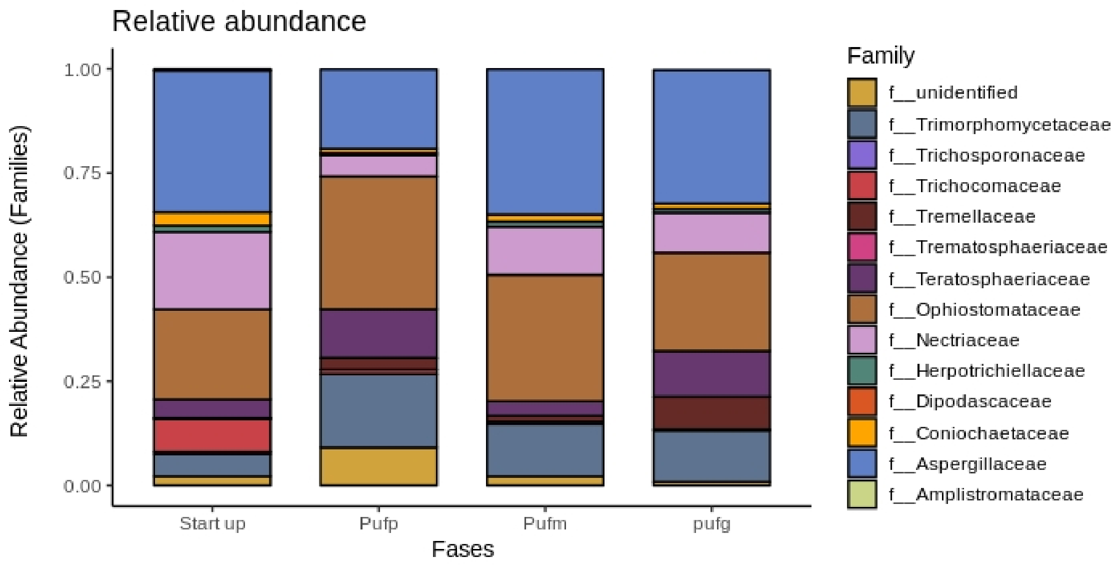


**(B)**

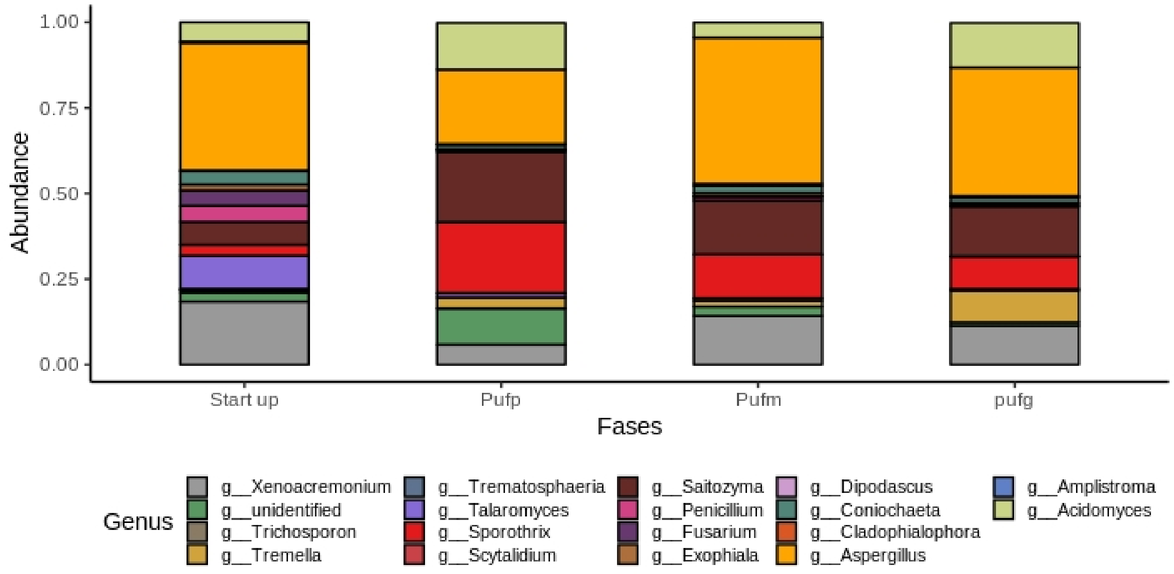


**Figure S14**. Fungal (**A**) families and (**B**) genera.
